# Single‐dose ibuprofen induced Stevens–Johnson Syndrome

**DOI:** 10.1002/ccr3.8574

**Published:** 2024-04-15

**Authors:** Ahmed Qasim Mohammed Alhatemi, Hashim Talib Hashim, Muhamad Abdulrahman Shyea Al‐Tarbosh, Rand Abdulhussain, Ali Talib Hashim

**Affiliations:** ^1^ Department of Internal Medicine Al Nasiriyah Teaching Hospital Thi Qar Iraq; ^2^ College of Medicine Warith Al Anbiyaa University Karbala Iraq; ^3^ Department of Pharmacy University of Huddersfield Huddersfield UK; ^4^ Golestan University of Medical Sciences Gorgan Iran

**Keywords:** acute medicine, critical care medicine, dermatology, immunology, pharmacology and pharmacy

## Abstract

**Key Clinical Message:**

Ibuprofen single dose may rarely induce Stevens–Johnson Syndrome, emphasizing the vital need for heightened vigilance in healthcare and public awareness for safer medication practices.

**Abstract:**

Stevens–Johnson Syndrome (SJS) is a severe and potentially life‐threatening skin disorder associated with certain medications, including ibuprofen. We present a case of a 45‐year‐old woman who developed SJS following a single dose of ibuprofen. Despite its rarity, this case underscores the importance of heightened vigilance in healthcare and public awareness regarding the potential risks of commonly used medications. Prompt recognition of SJS symptoms and immediate medical intervention are crucial for patient outcomes. Healthcare providers should exercise caution when prescribing ibuprofen, particularly in patients with a history of adverse drug reactions. This case emphasizes the need for ongoing monitoring, patient education, and informed decision‐making to promote medication safety and optimal patient care.

## Graphical abstract

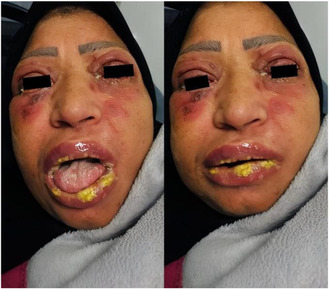

## 1 INTRODUCTION

Ibuprofen, a widely used nonsteroidal anti‐inflammatory drug (NSAID), has long been a stalwart in the realm of pain relief and inflammation management. However, behind its commonplace presence in medicine cabinets, there lies a potential peril that, though rare, demands our attention and understanding. This narrative unfolds as a middle‐aged individual embarks on an unexpected journey through the harrowing landscape of Stevens–Johnson Syndrome (SJS)—a severe and, at times, life‐threatening skin disorder.^1^, ^2^

As the story begins, it beckons us to consider the seemingly innocuous act of reaching for an over‐the‐counter pain reliever. In this case, ibuprofen serves as the unwitting protagonist, a common choice for those grappling with the discomforts of everyday ailments.^3^ The unsuspecting middle‐aged protagonist, seeking respite from a routine headache or muscular ache, unwittingly triggers a chain of events that will unfold into a rare and alarming medical ordeal.^4^

Stevens–Johnson Syndrome, characterized by the abrupt onset of a painful rash and blistering, often involves the mucous membranes, including the eyes, nose, and mouth. It can be triggered by various medications, and ibuprofen, despite its ubiquity and perceived safety, is not exempt from this list.^5^ The narrative explores the unfolding symptoms, the perplexing escalation from mild discomfort to severe skin involvement, and the subsequent medical odyssey that ensues.^5^

This exploration delves into the broader implications of drug safety, shedding light on the delicate balance between the benefits and risks of widely used medications. It underscores the importance of vigilance, both on the part of healthcare providers and on the public, in recognizing potential adverse reactions.^6^ Through this journey, we navigate the intricacies of drug‐induced skin disorders and the critical role of prompt medical intervention in steering the narrative toward recovery.^7^ “Ibuprofen Unveiled” invites readers to contemplate the uncharted territories that can emerge from seemingly routine decisions and prompts a reconsideration of our relationship with common medications that weave seamlessly into the fabric of our daily lives.

## 2 CASE PRESENTATION, HISTORY AND EXAMINATION

A 45 year‐old woman presented to the emergency department with a sudden onset of severe facial swelling, red painful eyes with discharge, crusting and erosion of lips, and a generalized non‐blanching purpuric rash, predominantly on the chest, upper, and lower limbs.

Upon thorough history‐taking, it was revealed that she had taken ibuprofen 800 mg single dose 4 h prior to admission for flu‐like symptoms 3 days ago. She had no past medical history of chronic diseases and was not taking any medications. She is a nonsmoker and nondrinker.

During examination, the patient, an overweight woman with a weight of 78 kg and a height of 163 cm and BMI of 29.4, appeared in distress but was fully conscious, oriented to time, place, and person. Cardiac, respiratory, abdominal, and neurological examinations were unremarkable. Her vital signs were as follows: BP 133/88 mmHg, RR 22, SpO_2_ 95%, HR of 88 bpm, and a temperature of 38.4°C.

**FIGURE 1:**
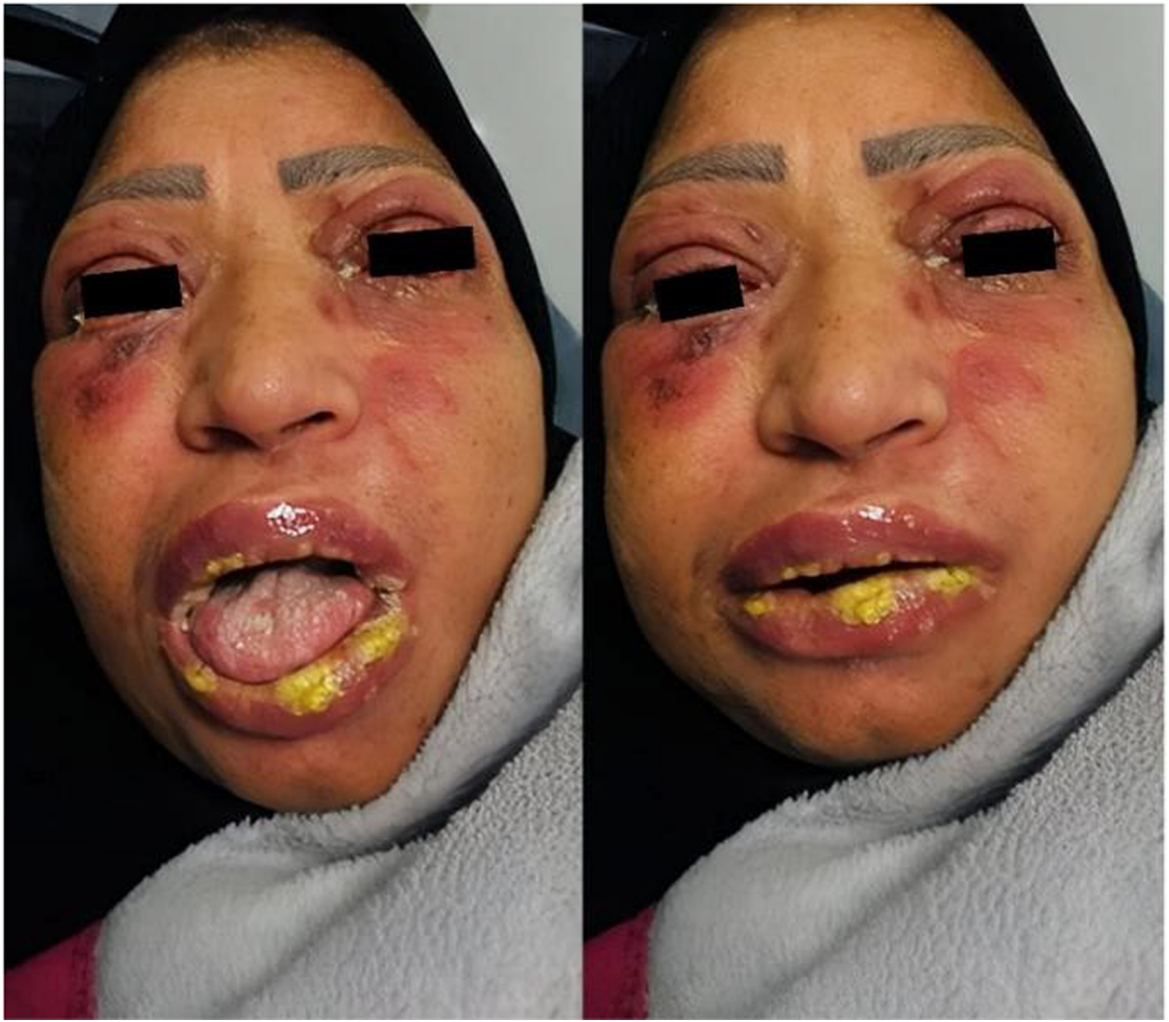
Conjunctivitis with discharge. Coalescing red erythematous macules with central blister formation in the face. Chelitis and yellowish discharge.

Head, neck, and eye examination revealed conjunctivitis with clear discharge, coalescing deep red erythematous targeted macules with central blister formation on the face, and swelling, erosion, and yellowish discharge of the lips (chelitis) as depicted in Figure 1.

**FIGURE 2:**
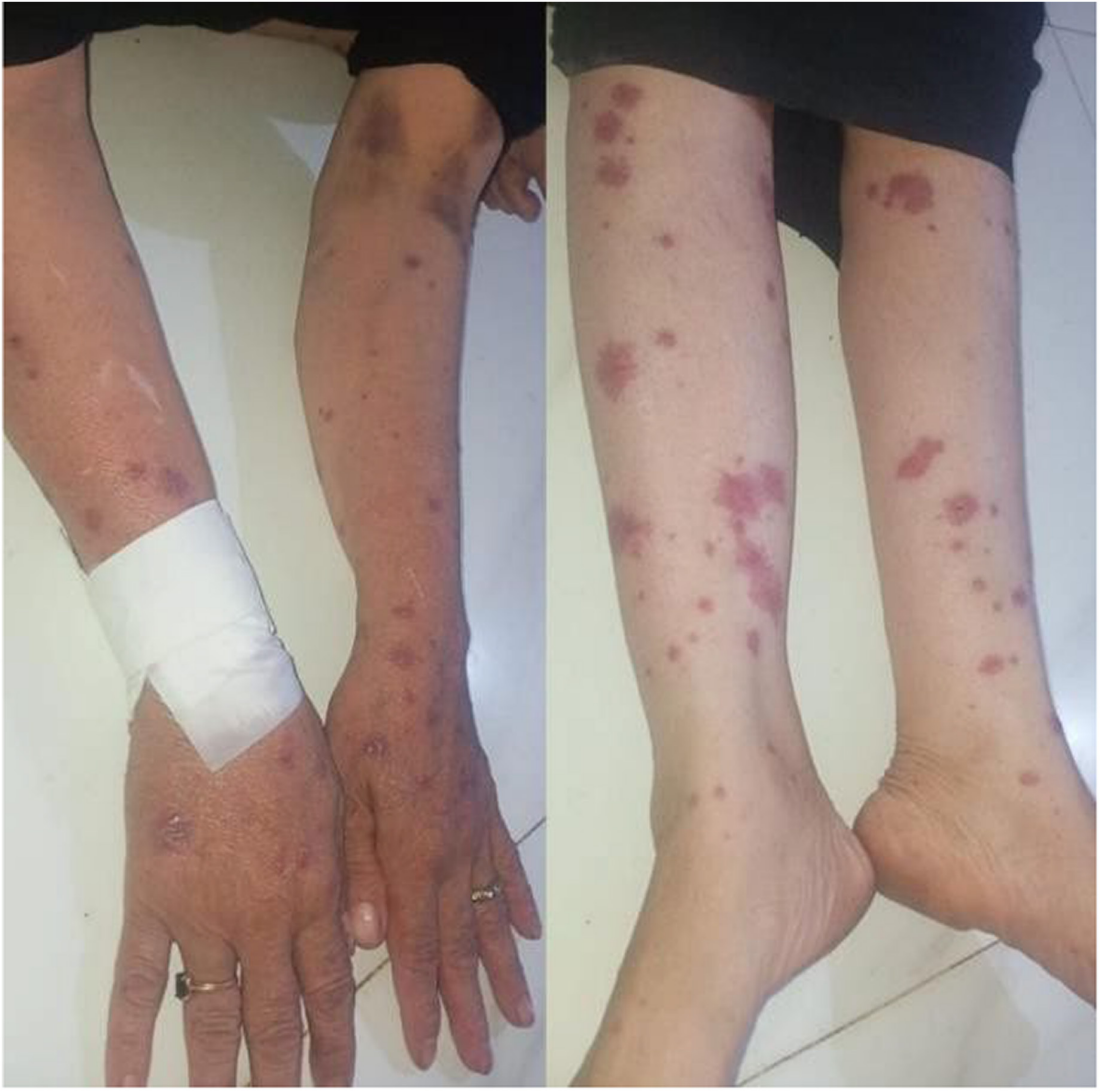
Nonblenching flat purpuric rash with target lesions in lower and upper limbs.

Musculoskeletal examination showed a nonblanching flat purpuric rash with target lesions on both upper and lower limbs with a percentage of body surface area (BSA) of 9%, illustrated in (Figure 2).

### 2.1 Deferential diagnosis, investigation, and management

Blood samples were collected for a complete blood count, inflammatory markers, renal and liver function tests, and serum electrolytes (Table 1).

**TABLE 1:** Blood tests showing anemia, leukocytosis, mild renal impairment, and elevated inflammatory markers.

Test	Results	Units	Normal value
Hb	11.2	g/dL	12–14
Wbc	15.9	10^10^/L	4–10
Plt	155	10^10^/L	150–400
B. Urea	56	mg/dL	15–40
S.	1.3	mg/dL	0.6–1.2
Creatinine			
CRP	56	mg/L	3–10
ESR	59	mm/h	0–30
ALT	50	U/L	10–130
AST	33	U/L	10–34
T. Bilirubin	0.4	mg/dL	0–0.8
S. Albumin	3.2	g/dL	2.4–4
PT	10.2	Seconds	9.5–12.5
INR	1.4		1–2
Sodium	139	mmol/L	135–145
Potassium	3.8	mmol/dL	3.5–5
Chloride	101	mmol/dL	96–106

**FIGURE 3:**
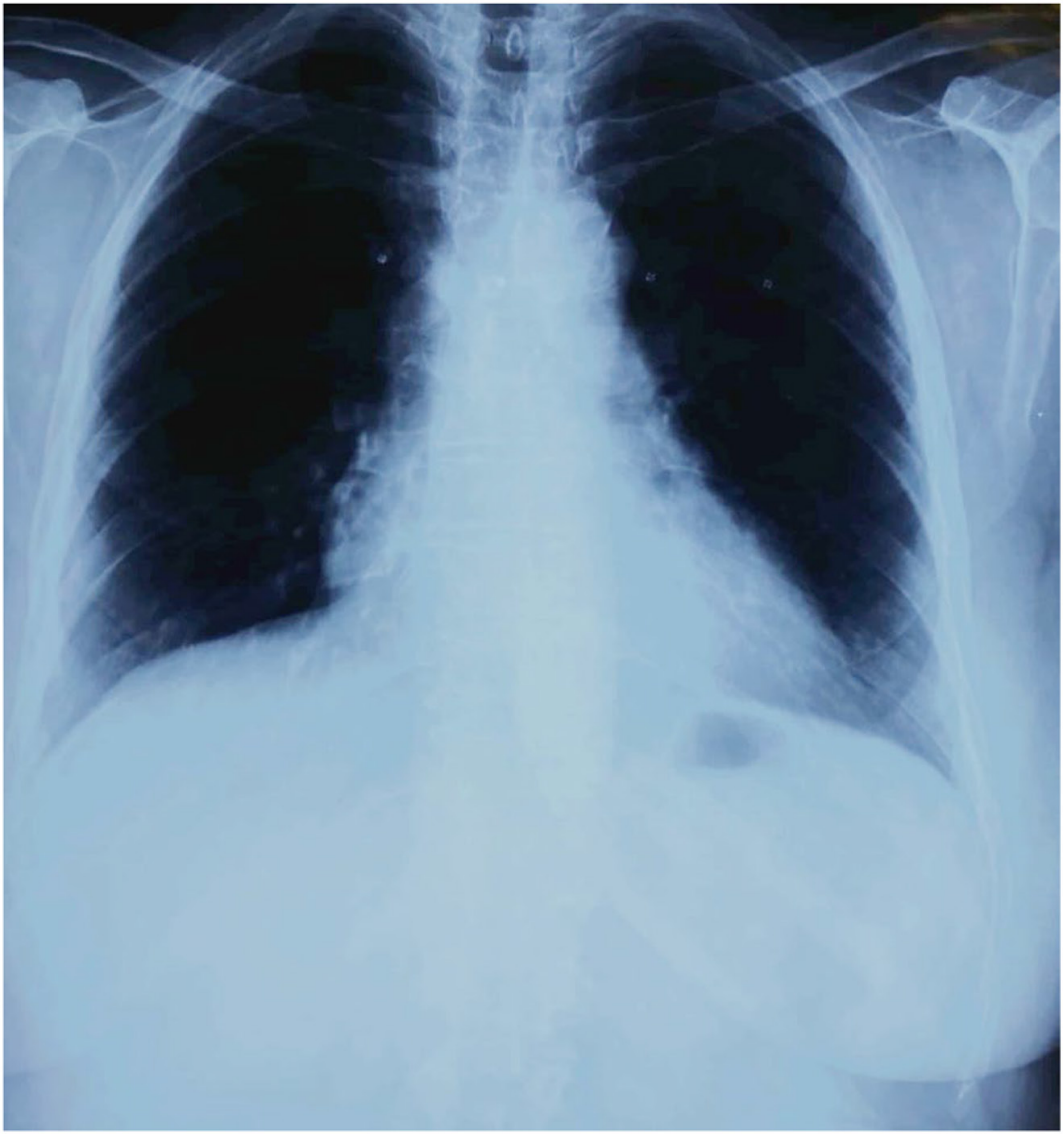
Initial Chest X‐ray PA view labeled as normal.

A comprehensive review of history and examination raised a high suspicion of SJS. Therefore, chest X‐ray (Figure 3) and testing for anti‐mycoplasma antibodies was conducted to rule out mycoplasma‐associated SJS. The results were negative.

**FIGURE 4:**
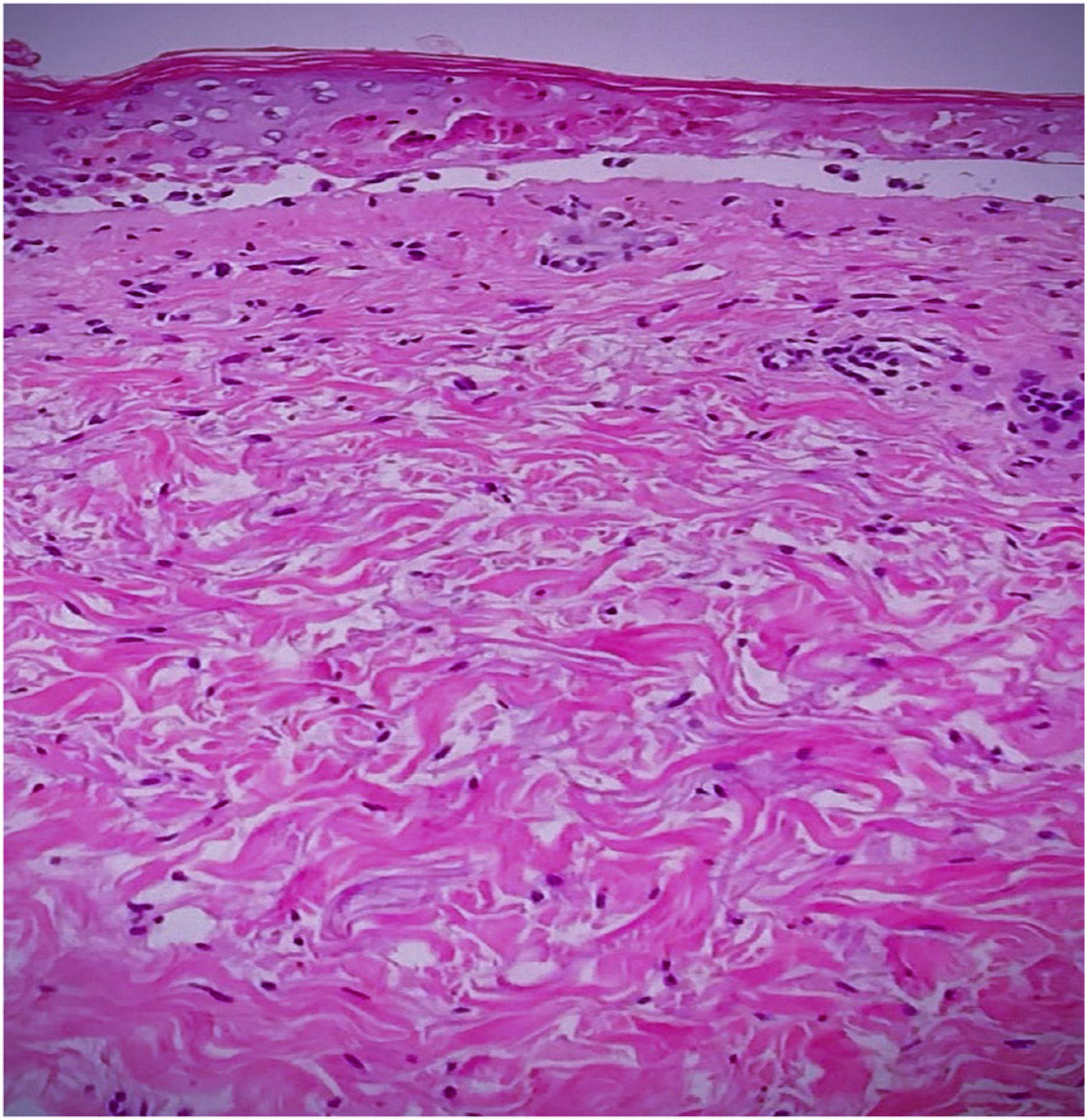
Skin biopsy histopathology specimen showing full‐thickness necrosis and separation of the epidermis at the dermoepidermal junction.

We proceeded further with a skin biopsy while admitting the patient for additional investigation and management. The result revealed full‐thickness necrosis and separation of the epidermis at the dermoepidermal junction, consistent with the diagnosis of SJS (Figure 4).

The patient was admitted to the Intensive Care Unit for strict management under aseptic conditions. Treatment included intravenous fluids, prophylactic antibiotics, lubricating eye drops, and dexamethasone 8 mg IV once‐daily for 7 days. A nasogastric tube was inserted on the second day to maintain oral hydration. Prophylactic heparin and omeprazole were implemented for the duration of hospitalization. Oral lesions were managed with mouthwashes and topical anesthetics.

## 3 CONCLUSION AND RESULTS

**FIGURE 5:**
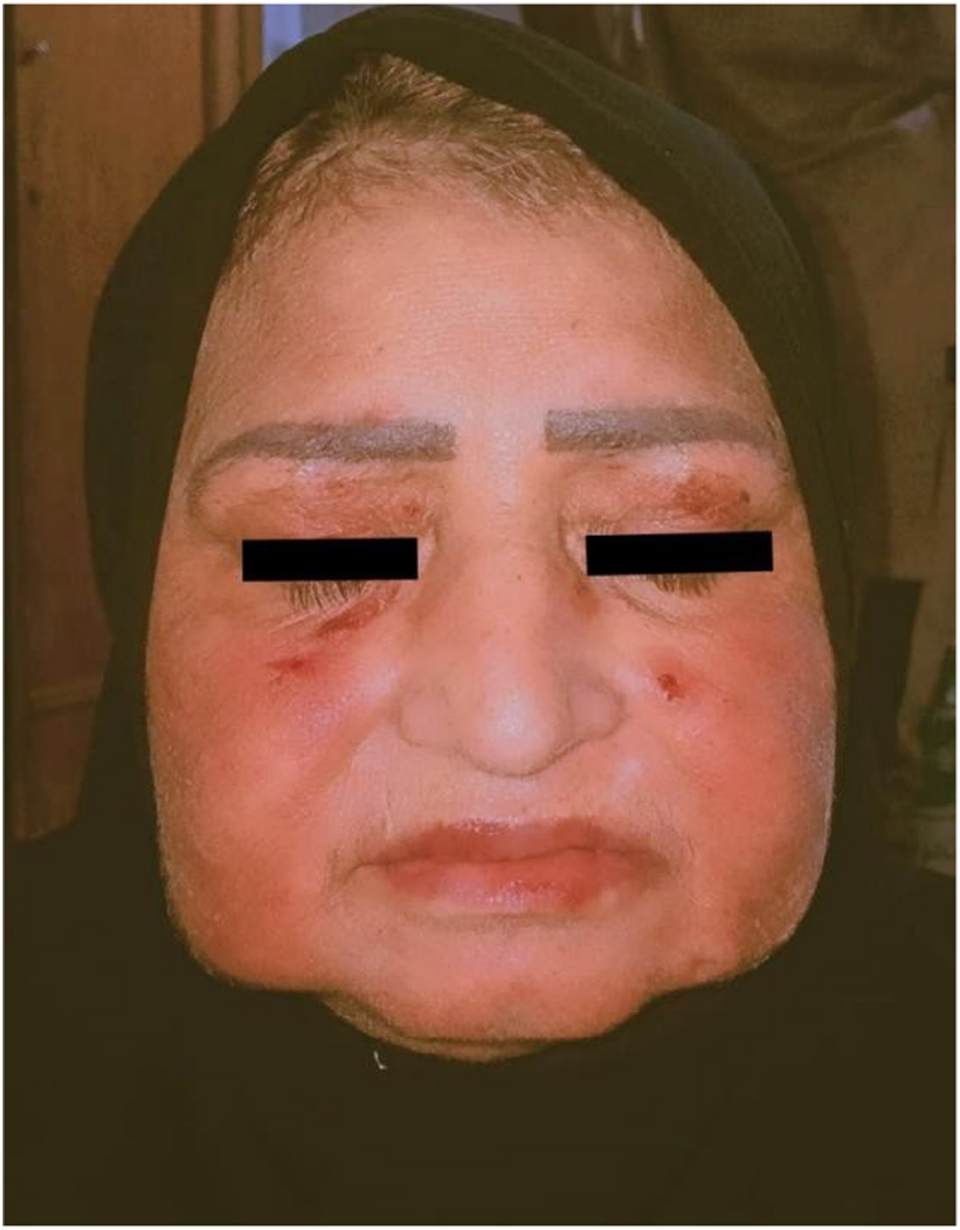
Decrease in size and color of facial rash with minimal ulcers and normal lips appearance 2 months after admission.

After 7 days of admission, the patient's condition showed significant improvement, prompting her discharge for regular follow‐up with a dermatologist, ophthalmologist, and internist. The facial and mucocutaneous rash and ulcers had notably decreased in severity, as depicted in Figure 5. This positive development reflects the effectiveness of the treatment regimen provided during her hospital stay. Continuing follow‐up appointments with the specialists was crucial to monitor her progress closely and ensure her ongoing recovery.

While ibuprofen is generally safe, the rare occurrence of severe reactions such as SJS serves as a reminder that no medication is entirely without risk. Middle‐aged individuals, like any other age group, should be vigilant about their health, be aware of potential side effects, and consult with healthcare professionals if they experience unexpected symptoms during ibuprofen use.

## 4 DISCUSSION

The existing discourse on the association between ibuprofen and SJS underscores its relative rarity among users of this widely utilized NSAID. While ibuprofen is generally regarded as safe for pain relief and inflammation, the emergence of severe adverse reactions, including SJS, introduces complexities that warrant closer examination.^8^

Stevens–Johnson Syndrome, characterized by a severe and painful rash leading to the detachment of the outer skin layer, typically manifests with abrupt onset and rapid progression, necessitating immediate medical attention.^9^ Notably, SJS is more commonly linked to certain infections and other medications, such as antibiotics and anticonvulsants, yet documented cases associated with ibuprofen use have been reported.

The unpredictability of severe drug reactions, as observed in middle‐aged individuals, challenges assumptions of reduced susceptibility in this demographic.^5^, ^7^ The idiosyncratic nature of the ibuprofen–SJS link, occurring in only a small percentage, adds complexity to understanding this adverse reaction. Genetic factors may contribute to predisposition, but the underlying mechanisms remain elusive.

An imperative facet is the consideration of single‐dose ibuprofen as a potential trigger for SJS, introducing a nuanced dimension to the discussion. While the linkage between ibuprofen and SJS remains incompletely understood, the distinctiveness of a single‐dose‐induced reaction emphasizes the need for heightened vigilance. This prompts a critical reevaluation of ibuprofen's safety profile, especially considering its widespread use in conditions such as arthritis and pain management.^10^

In navigating the intricate balance between ibuprofen's benefits and potential risks, healthcare providers must exercise heightened caution, particularly in individuals with a history of adverse reactions or known risk factors. Communication between patients and healthcare professionals becomes paramount for informed decision‐making concerning medication choices in light of the rare yet severe consequences associated with single‐dose ibuprofen‐induced SJS.

## 5 CONCLUSION

This case highlights the rare but serious risk of SJS following a single dose of ibuprofen. It underscores the importance of thorough history‐taking, symptom monitoring, and prompt medical intervention. Healthcare providers should exercise caution when prescribing ibuprofen, particularly in patients with a history of adverse drug reactions. Patient education about SJS symptoms is crucial for early detection and intervention. Vigilance, communication, and informed decision‐making are essential for promoting medication safety and optimal patient care.

## AUTHOR CONTRIBUTIONS

**Ahmed Qasim Mohammed Alhatemi:** Conceptualization; data curation; investigation; resources; visualization; writing – original draft; writing – review and editing. **Hashim Talib Hashim:** Conceptualization; data curation; investigation; visualization; writing – original draft; writing – review and editing. **Mohammed Abdul Rahman:** Conceptualization; data curation; formal analysis; resources; software. **Rand Abdulhussain:** Investigation; methodology; visualization; writing – original draft. **Ali Talib Hashim:** Investigation; methodology; project administration; software; supervision.

## FUNDING INFORMATION

We declare that no sources of funding were received.

## CONFLICT OF INTEREST STATEMENT

The authors have no conflict of interest to declare.

## CONSENT

Written informed consent was obtained from the patient to publish this report in accordance with the journal's patient consent policy.

## Data Availability

The data that support the findings of this study are available from the corresponding author upon reasonable request.
